# Biochemical functions and structure of *Caenorhabditis elegans* ZK177.8 protein: Aicardi–Goutières syndrome SAMHD1 dNTPase ortholog

**DOI:** 10.1016/j.jbc.2023.105148

**Published:** 2023-08-09

**Authors:** Tatsuya Maehigashi, Christopher Lim, Lydia R. Wade, Nicole E. Bowen, Kirsten M. Knecht, Natalie N. Alvarez, William G. Kelly, Raymond F. Schinazi, Dong-Hyun Kim, Yong Xiong, Baek Kim

**Affiliations:** 1 Department of Pediatrics, School of Medicine, Emory University, Atlanta, Georgia, USA; 2 Department of Molecular Biophysics and Biochemistry, Yale University, New Haven, Connecticut, USA; 3 Department of Biology, Emory University, Atlanta, Georgia, USA; 4 Center for ViroScience and Cure, Children’s Healthcare of Atlanta, Atlanta, Georgia, USA; 5 Neurobiota Research Center, College of Pharmacy, Kyung-Hee University, Seoul, South Korea

**Keywords:** SAMHD1, dNTPase, structure, *C. elegans*, ortholog

## Abstract

Mutations in sterile alpha motif domain and histidine–aspartate domain–containing protein 1 (SAMHD1) are found in a neurodevelopmental disorder, Aicardi–Goutières syndrome, and cancers, and SAMHD1, which is a deoxynucleoside triphosphate (dNTP) triphosphorylase, was identified as a myeloid-specific HIV-1 restriction factor. Here, we characterized the enzymology and structure of an SAMHD1 ortholog of *Caenorhabditis elegans*, ZK177.8, which also reportedly induces developmental defects upon gene knockdown. We found ZK177.8 protein is a dNTPase allosterically regulated by dGTP. The active site of ZK177.8 recognizes both 2′ OH and triphosphate moieties of dNTPs but not base moiety. The dGTP activator induces the formation of the enzymatically active ZK177.8 tetramers, and ZK177.8 protein lowers cellular dNTP levels in a human monocytic cell line. Finally, ZK177.8 tetramers display very similar X-ray crystal structure with human and mouse SAMHD1s except that its lack of the canonical sterile alpha motif domain. This striking conservation in structure, function, and allosteric regulatory mechanism for the hydrolysis of the DNA building blocks supports their host developmental roles.

A series of mutations in human sterile alpha motif (SAM) domain and histidine–aspartate (HD) domain–containing protein 1 (SAMHD1) gene were identified in Aicardi–Goutières syndrome (AGS) (1, 2, 3, 4). AGS is a neurodevelopmental genetic disorder with abnormally elevated innate immune functions including interferon and interferon-related gene expression in the absence of any detectable infection (1, 2), ultimately leading to abnormal brain development and death. Many genes mutated in AGS, such as SAMHD1, RNase H2, and Trex1, are involved in various cellular nucleic acid metabolisms, suggesting that the interrupted nucleic acid metabolisms may trigger cellular nucleic acid immune sensing, which potentially induces the activation of the cellular innate immune responses without any infection events (3, 4, 5).

SAMHD1 was also identified as a host restriction factor against lentiviruses such as HIV type 1 (HIV-1), type 2 (HIV-2), and simian immunodeficiency viruses (6, 7, 8). This antilentiviral activity of SAMHD1 is observed particularly in nondividing viral target cell types, including macrophages (6, 7, 8), dendritic cells (8), and resting T cells (9, 10, 11), which abundantly express SAMHD1. SAMHD1 harbors deoxynucleotide triphosphorylase (dNTPase) activity that hydrolyzes the building blocks of DNA and substrates of DNA polymerases, including reverse transcriptase, dNTP, into deoxynucleoside and triphosphate (12). We previously reported that human primary monocyte–derived nondividing macrophages harbor extremely limited cellular dNTP concentrations (20–40 nM), which is approximately 50 to 200 times lower than the dNTP concentration found in activated/dividing CD4+ T cells (2–5 μM) (13). This limited cellular dNTP availability in the nondividing cells kinetically restricts the reverse transcription step of HIV-1 (14, 15). Indeed, we also reported that SAMHD1 in terminally differentiated macrophages is responsible for the extremely low cellular dNTP concentration, which restricts lentivirus infection in this nondividing viral target cell types (13). However, unlike HIV-1, HIV-2 and many simian immunodeficiency viruses encode an accessary protein, viral protein X (Vpx), which proteosomally degrades SAMHD1 (7, 8), leading to the elevation of the cellular dNTP levels and ultimately to the infectivity enhancement of these lentiviruses in the myeloid cells (16).

The dNTPase activity of SAMHD1 lies at the C–T catalytic HD domain, and the N–T SAM domain is dispensable for the dNTPase activity of SAMHD1 (7, 12, 17). The dNTPase activity of SAMHD1 is regulated by two allosteric sites, A1 and A2 (18, 19, 20): dGTP/GTP bind to the A1 site, and dNTPs bind to the A2 site. The binding of these nucleotides triggers the formation of the enzymatically active SAMHD1 tetramers (21, 22). The structure of the full-length SAMHD1 protein is not currently available, whereas the HD domain structures of human SAMHD1 (hSAMHD1) revealed regulatory coordination between allosteric sites and the dNTPase active site in the enzymatically active tetramer form. hSAMHD1 dNTPase activity is also negatively regulated by phosphorylation at T592 residue near its C–T end, and SAMHD1 mainly stays as phosphorylated form in dividing cells (23). Current models suggest that the phosphorylation at T592 prevents the formation of the enzymatically active SAMHD1 tetramers (24, 25). Several previous studies also reported that SAMHD1 has nuclease and nucleic acid–binding activities (26, 27, 28, 29), whereas the roles of these activities of SAMHD1 need to be further investigated (30).

A series of recent studies reported that various SAMHD1 mutations are also found in multiple human cancers such as acute myeloid leukemia (31, 32), chronic lymphocytic leukemia (33, 34), and colon cancer (35, 36), supporting its potential roles in cell proliferation through the upregulation of intracellular dNTPs that is a well-known biochemical marker of cancer cells (37). Indeed, an SAMHD1 knockout study revealed that SAMHD1 controls cell cycle status and apoptosis in a myeloid cell line (38), and many mutations found in cancer cells induce the loss of its dNTPase activity (39). Furthermore, SAMHD1 is also involved in dsDNA break repair even though this activity does not require the dNTPase activity (40, 41). SAMHD1 also controls the nascent DNA processing activity of Mre11 nuclease at DNA replication forks, which may avoid interferon induction during DNA replication stress (42). Finally, it was reported that SAMHD1, which also hydrolyzes ara-CTP (43), is a marker for the ara-C treatment of acute myeloid leukemia patients (44).

SAMHD1 proteins are highly conserved among mammals including mice, and SAMHD1 orthologs can also be found in invertebrates. However, two independent studies with SAMHD1 knockout mice failed to display the AGS phenotypes even though these knockout mice exhibited the elevations of both interferon-response gene expression and cellular dNTP levels in various tissues (3, 45). While, in zebrafish, SAMHD1 is reportedly involved in the brain developmental process (46); currently, invertebrate animal models, such as *Caenorhabditis elegans* (*C. elegans*) and *Drosophila*, which are highly useful genetic platforms to dissect fundamental cellular and molecular function of gene homologs and orthologs, are not available for AGS.

Importantly, a previous genome-wide survey of *C. elegans* with RNA knockdown technology reported that the reduced expression of SAMHD1 ortholog, ZK177.8, led to maternal sterility (47), implying that ZK177.8 is also involved in host early development as observed with SAMHD1 in AGS. However, ZK177.8 contains only the C–T dNTPase–related HD domain without the SAM domain–like sequences in its N–T region. Furthermore, another SAMHD1 ortholog in fruit fly, *falten*, also lacks full N–T SAM domain, and the fruit fly with defects in the *falten* gene also present developmental problems, particularly during gastrulation stage (48). Since *C. elegans* has been extensively developed as animal models for human diseases, and it is possible that ZK177.8 can also carry those multiple *in vivo* functions of SAMHD1 found in human diseases, it is crucial to validate functionally the SAMHD1 ortholog protein in order to develop this nematode system to proper animal models. Here, we report that ZK177.8 protein is a dGTP-dependent dNTPase with very similar enzymatic and structural properties with hSAMHD1, supporting the conserved developmental role of this SAMHD1 ortholog in the invertebrate *C. elegans* as observed with SAMHD1 in vertebrates including humans.

## Results

### *C. elegans* ZK177.8 protein

**Figure 1:**
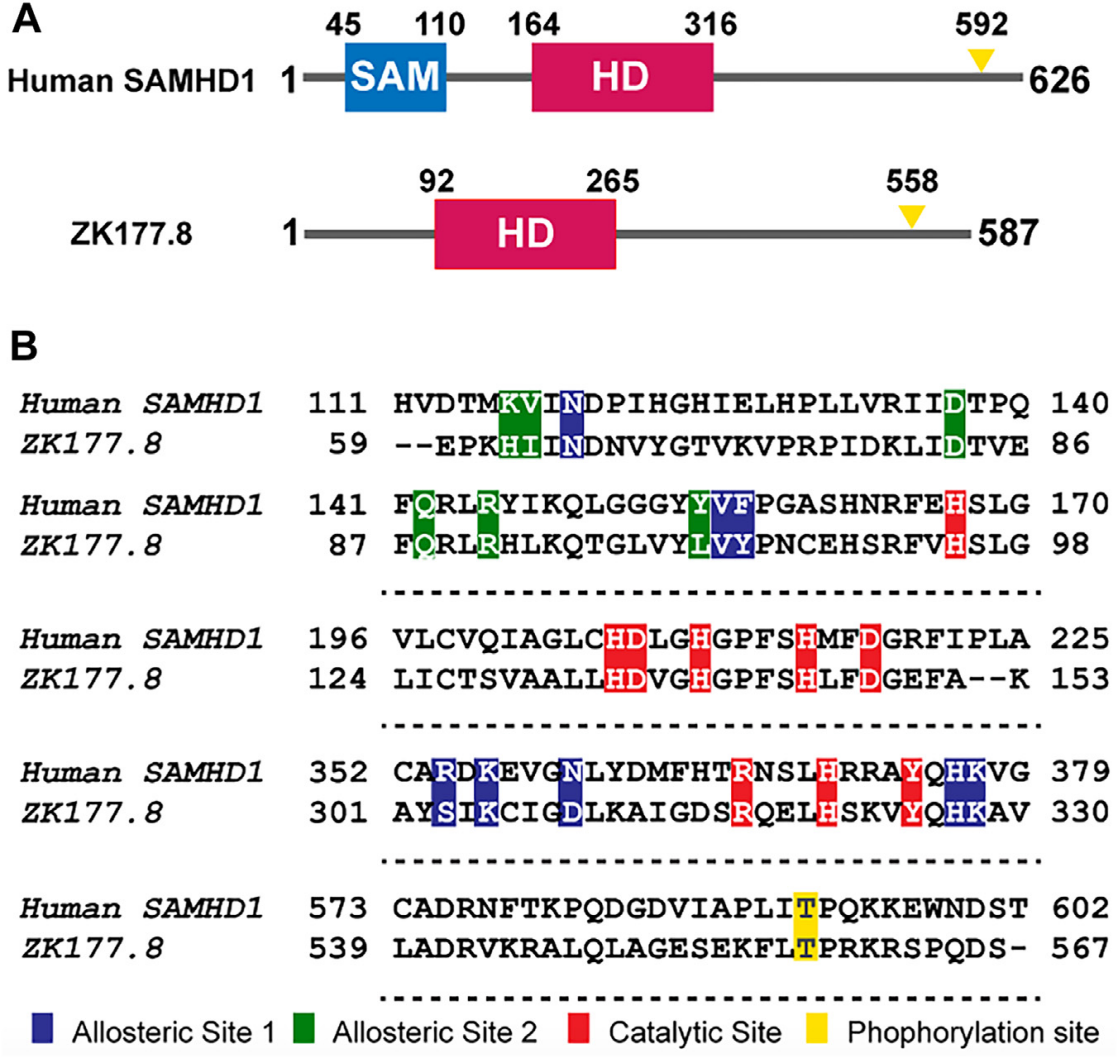
**Gene structure and amino acid sequence comparison.***A*, gene structure diagram: N–T SAM domain (*blue*), central HD domain (*red*), and C–T phosphorylation regulatory domain (*yellow*) are marked. Amino acid numbers of each functional domain are also marked. ZK177.8 lacks identifiable SAM domain–like sequence. *B*, amino acid sequence comparison in HD domain regions of human SAMHD1 and ZK177.8: allosteric site 1 (*blue*), allosteric site 2 (*green*), catalytic site (*red*), and phosphorylation site (*yellow*) were marked. Amino acid numbers of each protein were also marked. HD, histidine–aspartate; SAM, sterile alpha motif; SAMHD1, SAM domain and HD domain–containing protein 1.

The coding region of the *C. elegans* ZK177.8 gene (Fig. 1*A*) encodes 567 amino acid protein that contains the dNTPase-related HD domain with a series of highly conserved amino acid residues found in two separate allosteric regulatory sites (A1—*blue* and A2—*green* in Fig. 1*B*) as well as dNTPase catalytic site (*red*) of hSAMHD1. Also, ZK177.8 encodes a conserved threonine residue at its C–T end (Fig. 1*B*, *yellow*), which is known to be a phosphorylation site for the negative regulation of the dNTPase activity in hSAMHD1 (49). However, as illustrated in Figure 1*A*, the N–T region of ZK177.8 is much shorter than hSAMHD1 without any SAM domain–like sequences. For hSAMHD1, the SAM domain is dispensable for its dNTPase activity (17).

In order to enzymologically characterize the ZK177.8 gene product, the 567 amino acid long full-length ZK177.8 gene was amplified by RT–PCR from cellular RNAs isolated from adult *C. elegans*. The amplified gene was cloned to pGEX5x-3 for overexpressing the N–T glutathione-*S*-transferase (GST)-fused ZK177.8 protein in *Escherichia coli*. The overexpressed GST-fused ZK177.8 protein was initially applied to the GST affinity column, and the GST-free ZK177.8 protein was eluted after the treatment with factor Xα protease on-column that cleaves the sequence between GST tag and ZK177.8 protein. The GST-free ZK177.8 protein was further purified by gel filtration chromatography (Fig. S1*A*). We also constructed and purified a mutant of ZK177.8 containing mutated highly conserved HD residues (H134R/D135N) at the conserved dNTPase catalytic site, which are equivalent to H206 and D207 in hSAMHD1. These purified ZK177.8 proteins exhibited the expected molecular weight, which is smaller than hSAMHD1 because of lack of the SAM domain; however, they appear predominantly as dimers when compared with gel filtration standards (Fig. S1*B*). In this study, the dimer fractions were pooled for the biochemical characterization.

### Test for dNTPase activity of ZK177.8 protein

**Figure 2:**
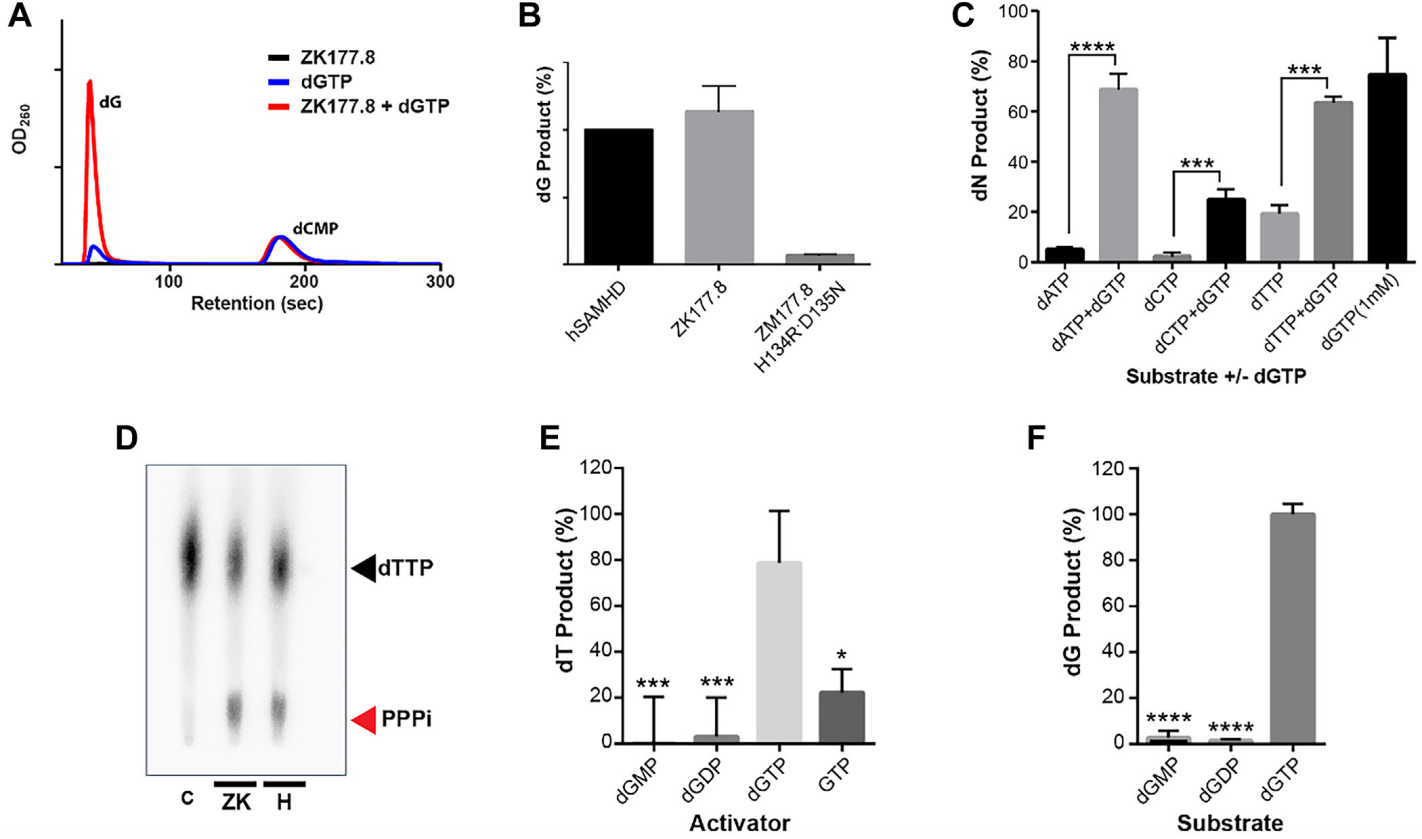
**ZK177.8 dNTP triphosphohydrolase activity and its activators.***A*, HPLC-based dNTP hydrolysis assay to detect deoxynucleoside (dN) product. dGTP substrate (1 mM) was incubated with purified ZK177.8 protein (1 μM) under the standard reaction condition including dCMP as loading control. The reactants were applied to HPLC for the detection of the dG product (dG). *Blue*: no ZK177.8 protein control, *red*: dGTP with ZK177.8, and *black*: ZK177.8 without dGTP. *B*, verifying catalytic inactivity induced by mutations H134R and D135N that are equivalent to those found in hSAMHD1 catalytic mutants (H206R and D207N). Determined by the HPLC assay, and dG products (%) are shown. *C*, dGTP-dependent dNTP triphosphorylase activity. dATP, dCTP, and dTTP were incubated with ZK177.8 protein individually under the standard reaction condition in the presence and absence of dGTP (50 μM). The reactants were applied to the HPLC-based assay to determine the normalized percent of dN products relative to dCMP loading control. dGTP alone was incubated for control. *D*, TLC-based dNTP hydrolysis assay to detect the triphosphate product (PPPi): α-^32^P-labeled dTTP was incubated with purified ZK177.8 (ZK) or human SAMHD1 (H) proteins under the standard condition including dGTP activator (see the Experimental procedures section), and the reactants were applied to TLC for the separation of PPPi from dTTP substrate. C: no enzyme control. *E*, test for the activator specificity of ZK177.8. The dTTP (1 mM) hydrolysis activity of ZK177.8 protein was conducted in the presence of activators, dGMP, dGTP, dGTP, and GTP (50 μM), and the dT products were determined by the HPLC assay. *F*, HPLC-based analysis for the hydrolysis of deoxyguanosine nucleotides with various phosphate length ZK177.8. dG product (%) is shown. dNTP, deoxynucleoside triphosphate.

Next, we tested whether the purified ZK177.8 protein acts as a dNTPase by using two different assays. First, we used the HPLC-based dNTPase assay for ZK177.8, which quantitatively determines the dN product generated from the hydrolysis of dNTP. We incubated dGTP (1 mM) with ZK177.8 protein (1 μM), and the products were applied to HPLC. As shown in Figure 2*A*, the dG product was detected in the reaction with ZK177.8 protein. However, when dGTP was incubated with the ZK177.8 HD catalytic site mutant (H134R/D135N), dG product was not detected (Fig. 2*B*). Next, we tested whether ZK177.8 also used dGTP as an allosteric activator for dNTPase activity as observed with hSAMHD1. As shown in Figure 2*C*, in the reactions with dATP, dCTP, or dTTP substrate, the respective dN products were observed in the presence of dGTP but not in the absence of dGTP. Next, we employed TLC-based dNTPase assay that can detect the triphosphate product. This assay uses α-^32^P labeled dTTP substrate, generating the radiolabeled triphosphate (PPPi) product after hydrolysis, which can be separated in TLC. As shown in Figure 2*D*, the incubation of the radiolabeled dTTP with 1 μM of ZK177.8 or hSAMHD1 in the presence of dGTP generated high levels of the radioactive TP product, compared with no enzyme control reaction that showed no detectable TP level. Next, since hSAMHD1 can also use rGTP for its activator (18), we quantitated the dTTP hydrolysis in the presence of rGTP, and ZK177.8 was also able to hydrolyze dATP in the presence of rGTP (Fig. 2*E*). Next, we tested whether dGMP and dGDP can work as A1 allosteric activators of ZK177.8 by using dTTP as a substrate. As shown in Figure 2*E*, dGMP and dGDP showed much limited capability to activate ZK177.8 to hydrolyze dTTP, compared with dGTP, which was previously observed with hSAMHD1 (18). These data support that the A1 site of ZK177.8 requires the triphosphate moiety of the activator for its full activation capability. Finally, we tested whether dGMP and dGTP can be hydrolyzed by ZK177.8 in the presence of the rGTP activator. As shown in Figure 2*F*, ZK177.8 could not hydrolyze dGMP and dGDP, which likely results from the failure of dGMP and dGDP in binding to the A2 activator site or triphosphorylase active site of ZK177.8. Overall, Figure 2 demonstrates that ZK177.8 harbors dGTP/GTP-dependent allosteric dNTP triphosphorylase activity that produces dN and TP, as observed with hSAMHD1 protein (12, 50).

### Substrate specificity of ZK177.8

**Figure 3:**
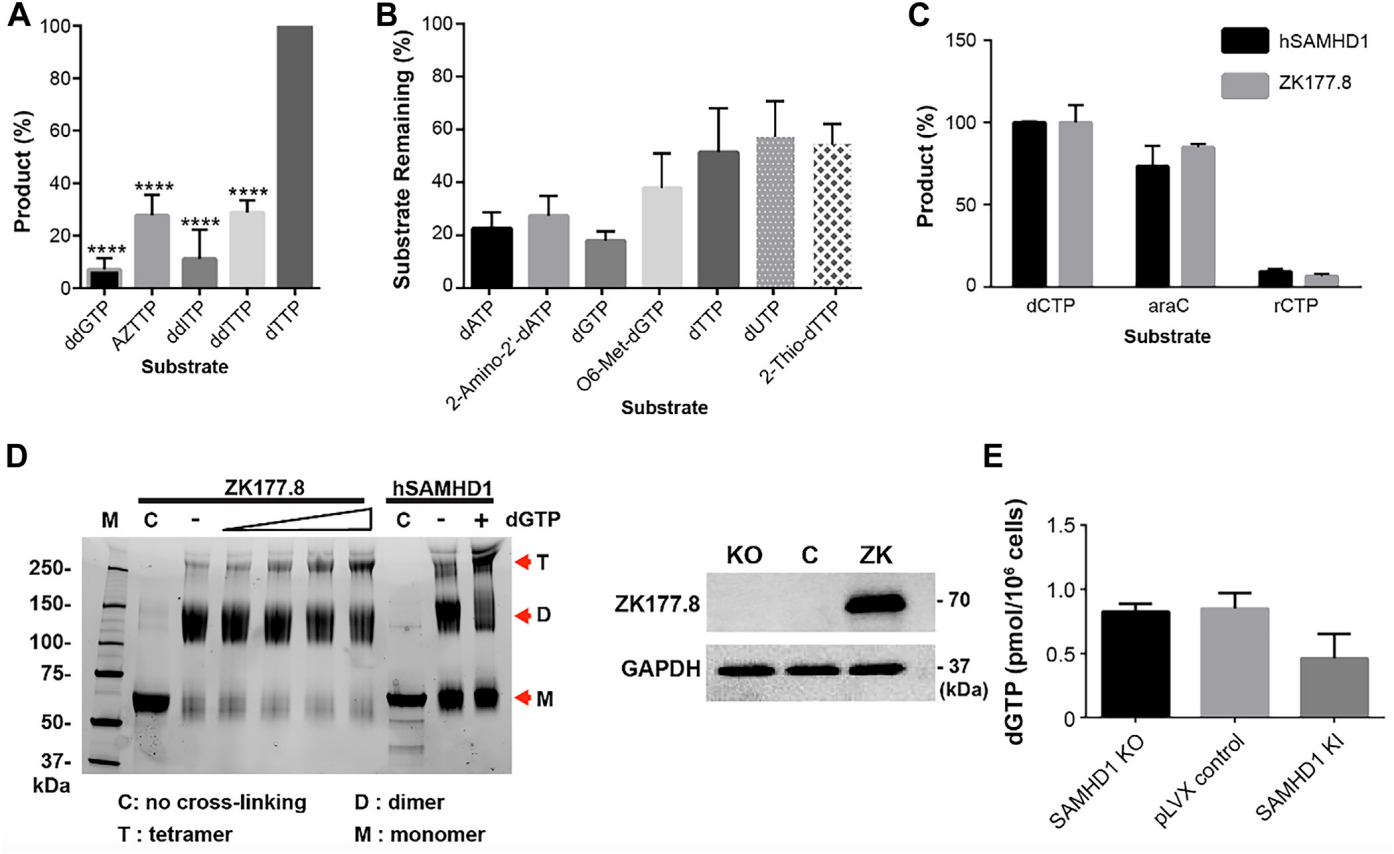
**Substrate specificities, oligomeric states, and cellular dNTP regulation of ZK177.8 protein.***A*, test for the hydrolysis of dNTP analogs lacking 2′ OH. ddGTP, AZTTP, dITP, and ddTTP (1 mM) were individually incubated with ZK177.8 protein under the standard reaction condition (50 μM dGTP activator), the corresponding nucleoside products were determined by the HPLC-based assay, and dTTP was used as a positive control. *B*, test for the hydrolysis of dNTP substrates with base modifications: 6-methy-dGTP, 2-amino-2′-dATP, 2-thio-dTTP, and dUTP (1 mM). Their corresponding unmodified dNTPs were used for comparison with 50 μM dGTP activator. O6-Methyl-dGTP hydrolysis was also compared with dGTP hydrolysis by ZK177.7. All dNTPase assays were conducted in triplicates. *C*, test for hydrolysis of nucleotides with 2′-OH: araC (arabionofuranosylcytosine triphosphate) and rCTP differ by their 2′-OH orientation, where the former takes the (2′S)-2′-OH (“down”) and the latter is (2′R)-2′-OH (“up”) configuration. HPLC-based analysis was used to measure product formation (%) of these analogs (1 mM). dCTP (1 mM) was used as positive control. *D*, dGTP-dependent oligomerization of ZK177.8. ZK177.8 protein (10 μM) was incubated in the presence of different concentrations of dGTP (0, 0.1, 0.4, 1, and 2 mM) and then crosslinked by formaldehyde before applied to SDS-PAGE as previously reported (60). Human SAMHD1 was also crosslinked with (+) and without (−) of 2 mM dGTP preincubation. C: no dGTP control. M: monomer, D: dimer, T: tetramer. Molecular weight markers were labeled (kilodalton). *E*, effect of ZK177.8 expression on cellular dNTP levels in differentiated/nondividing human monocytic THP-1 cells lacking hSAMHD1. THP-1 SAMHD1 KO cells were transduced with a lentivirus c-expressing HA-tagged ZK177.8 and mCherry protein as well as empty vector expressing only mCherry protein, and the mCherry protein–expressing cells were FACS sorted and propagated. The propagated cells were differentiated to nondividing macrophages by PMA for 7 days, and the dGTP levels in these cells were quantified by RT-based dNTP assay (13). dNTP, deoxynucleoside triphosphate; FACS, fluorescence-activated cell sorted; HA, hemagglutinin; hSAMHD1, human SAM domain and HD domain–containing protein 1; PMA, phorbol 12-myristate 13-acetate.

It was previously reported that hSAMHD1 fails to hydrolyze dNTP chain terminators lacking 3′ OH, which enhances the antiviral activity of these chain terminators particularly in macrophages (12). First, we examined whether ZK177.8 hydrolyzes the chain-terminator triphosphates lacking both 2′ and 3′ OHs, including ddGTP, AZTTP, ddITP, ddTTP, in the presence of dGTP activator. As shown in Figure 3*A*, ZK177.8 displayed significantly reduced capability to hydrolyze these chain terminator triphosphates, suggesting that ZK177.8 also requires 3′ OH for its full dNTPase activity. Next, we tested whether ZK177.8 recognizes the base moiety of dNTP substrates. As shown in the reactions with 2′-amino-2′-dATP, O6-methyl dGTP, dUTP, and 2-thio dTTP (Fig. 3*B*), ZK177.8 hydrolyzed these dNTPs with base modifications with similar efficiency with their natural dNTP partners. Finally, while hSAMHD1 does not degrade rNTPs (12), a series of recent studies demonstrated that hSAMHD1 hydrolyzes an anticancer agent (43, 51), arabinofuranosylcytosine triphosphate, which still has 2′ OH but in “up” position (2′S-OH) as well as 3′ OH. As shown in Figure 3*C*, ZK177.8 was able to hydrolyze arabinofuranosylcytosine triphosphate but not ribocytidine triphosphate. These data support that ZK177.8 recognizes its dNTP substrates in a very similar pattern with hSAMHD1 (43).

### Tetramerization of ZK177.8 protein

The dGTP binding to both A1 and A2 sites triggers the formation of the enzymatically active tetramers of hSAMHD1 (21, 22). Next, we tested the tetramerization formation of ZK177.8 by dGTP. For this test, we conducted formaldehyde-induced crosslinking of ZK177.8 in the presence and absence of dGTP, which was previously used for detecting the hSAMHD1 tetramerization (21, 22). As shown in Figure 3*D*, we detected the tetramer formation of hSAMHD1 protein only in the presence of dGTP. Indeed, we also observed the increased levels of the ZK177.8 tetramers when the dGTP concentration was gradually elevated, whereas no ZK177.8 tetramer form was detected in the absence of dGTP (“C” in ZK177.8, Fig. 3*D*). These data support that dGTP allosteric activator mediates the formation of the enzymatically active ZK177.8 tetramers.

Next, we tested whether ZK177.8 protein affects cellular dNTP levels in human cells. For this test, we employed a human monocytic SAMHD1 knockout THP-1 cell line that we previously established by CRISPR–Cas9 (38). Upon differentiation to nondividing THP-1 macrophages by phorbol 12-myristate 13-acetate (PMA) treatment, the dNTP levels in this SAMHD1 knockout THP-1 cell line increased by threefold to fivefold, compared with both parental THP-1 cells and the control cells transduced with the empty knockout control vector, suggesting that the loss of hSAMHD1 elevates cellular dNTP levels in nondividing THP-1 cells (38). For this test, first, we expressed ZK177.8 protein in the THP-1 SAMHD1 KO cells using the pLVX-IRES-mCherry lentiviral vector system expressing both hemagglutinin (HA)-tagged ZK177.8 and mCherry protein or only mCherry protein (control). The mCherry+ cells were fluorescence-activated cell sorted and propagated. Next, the sorted mCherry+ cells were analyzed for the ZK177.8 expression by Western analysis with HA antibody after PMA differentiation to the nondividing macrophage stage. As shown in Figure 3*E*, ZK177.8 protein was expressed in the hSAMHD1 KO THP-1 cells transduced with the pLVX vector expressing both ZK177.8 and mCherry protein but not in the control cells transduced with the empty pLVX vector expressing only mCherry protein. When we measured the dGTP levels in these THP-1 cells after differentiation to the nondividing macrophage stage by PMA (Fig. 3*F*), the SAMHD1 KO THP-1 macrophages expressing ZK177.8 and mCherry protein displayed reduced dGTP level, compared with the SAMHD1 KO cells that express only mCherry protein, suggesting that ZK177.8 can hydrolyze cellular dNTPs in the PMA-differentiated nondividing THP-1 cells lacking hSAMHD1.

### X-ray crystal structure of ZK177.8

**Figure 4:**
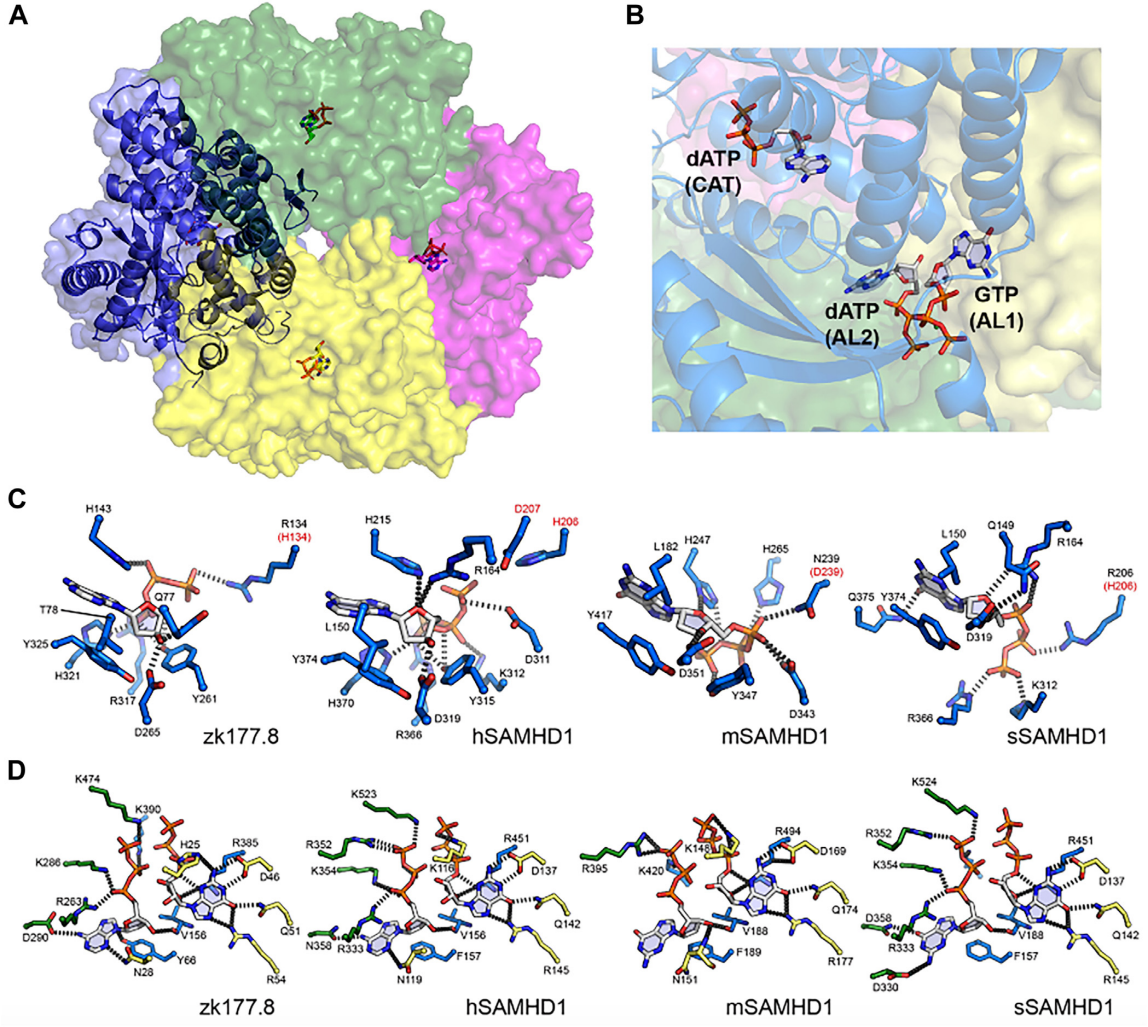
**Structure of ZK177.8 by X-ray crystallography.***A*, overall representation of ZK177.8 tetramer shown in transparent surface, with one monomer in *cartoon* and nucleotides (dATP) bound in catalytic site in each monomer in *stick* representation. The view is oriented down on a twofold axis. *B*, respective locations of catalytic site ligand (dATP) and two allosteric site ligands (GTP for A1 and dATP for A2) are shown in *stick* model. Allosteric sites are located at “tetramer interface,” where two dimers contact. *C*, detailed analysis of catalytic sites of ZK177.8 and SAMHD1 orthologs. A bound nucleotide is shown at center, with key interacting side chains shown in *sticks*, with hydrogen bondings indicated by *dotted line*. *D*, detailed view of allosteric sites of ZK177.8 and SAMHD1 orthologs. In each subpanel, AL1 site–bound nucleotide is shown in *right*, and AL2 site–bound nucleotide is shown on *left*. As in (*C*), key interacting side chains are also shown in *sticks*, with H-bonds indicated by *dotted line*. All figures were rendered with the program PyMOL (65). SAMHD1, SAM domain and HD domain–containing protein 1.

Next, we solved the crystal structure of ZK177.8 HD domain–containing catalytic core construct of ZK177.8 (residues 41–565, ZK177.8^41–565^ hereafter), which was purified employing the same protocol as used for the full-length protein. ZK177.8^41–565^ was crystallized in space group P1. A total of 198,955 unique reflections in the resolution range 1.8 to 42.5 Å were used in the refinement. The final *R*-factor is 18.2% and *R*_free_ is 20.9% (Table 1). The asymmetric unit contains a tetramer of ZK177.8^41–565^ (Fig. 4*A*) with D2 (222) symmetry, which is the active form of the biological assembly. This tetrameric form of active SAMHD1 structure has been observed in all other SAMHD1 orthologs from mouse and pig (52, 53).

**Table 1:** Data collection and refinement statistics

	ZK177.8
Space group	P 1
Resolution (Å)	46.03–1.81 (1.87–1.81)
Cell dimensions	
a, b, c (Å)	90.68, 90.56, 93.36
α, β, γ (°)	65.59, 65.49, 86.22
*R*_merge_	0.098 (0.67)
*CC*_*1/2*_	0.998 (0.237)
Mean I/sigma (I)	7.9 (1.2)
Completeness (%)	94.2 (91.5)
Redundancy	2.2 (2.2)
Refinement	
*R*_work_/*R*_free_	0.21/0.18 (0.33/0.35)
No. of atoms	
Protein	16,303
Ligand	420
Water	1482
*B*-factors	
Protein	30.4
GTP	14.6
dATP	27.7
Water	38.2
RMSD	
Bonds (Å)	0.017
Angles (º)	1.9
Ramachandran statistics	
Favored (%)	98.3
Allowed (%)	1.7
Outliers (%)	0.0

One crystal was used for data collection and structure determination. Statistics for the highest-resolution shell are shown in parentheses.

Our model shows that each monomer of ZK177.8 HD domain consists of a total of three nucleotides bound (Fig. 4*B*). First, within each monomer, a dATP molecule occupies its catalytic site. The crystallographic construct used here contains catalytic mutations of H134R/D135N that are equivalent to catalytically inactive mutations of H205R/R206N in hSAMHD1; therefore, the dATP substrates occupying its catalytic sites are at unhydrolyzed state, and no catalytic metal ions are observed in the reported structures (Fig. 4*C*). Our three-dimensional homology searches using Dali server also returns high structural similarities to other SAMHD1 orthologs, including *Mus musculus* (mouse) SAMHD1 (mSAMHD1) (52) (Protein Data Bank [PDB] ID: 6BRK, *z*-score of 34.2, RMSD of 2.4 Å, and sequence identity of 32%) and *Sus scrofa* (pig) SAMHD1 (sSAMHD1) (53) (PDB ID: 5YHW, *z*-score of 47.4, RMSD of 1.6 Å, and sequence identity of 35%) that has been released to the PDB. Indeed, a careful comparison between our model and other SAMHD1 orthologs reveals that many key residues interacting with the nucleotide are nearly identical (Fig. 4*C*). For example, the presence of the tyrosine residue near 2′ position of the ribose, which is presumably responsible for the dNTP selection for hydrolysis (21, 43), is also seen in ZK177.8 (Tyr325) at a very similar position as in other orthologs compared here (Tyr374 in hSAMHD1 and sSAMHD1 and Tyr417 in mSAMHD1) (Fig. 4*C*). As expected from sequence alignment (Fig. S2), another residue responsible for contributing to the tight catalytic pocket, Leu150 in hSAMHD1 (21), is substituted by a smaller side-chain residue Thr78 in ZK177.8. Specifically, Leu150 in hSAMHD1 has been proposed to be responsible for the substrate selection against (2′R)-2′-F and (2′R)-2′-OH nucleotides with 2′-OH group in “up” position, including rNTPs (43, 52), mainly because of its steric hindrance that disfavors the binding of these molecules. Therefore, we speculated an altered profile in terms of substrate specificity with smaller side-chain substituent in ZK177.8. However, the direct overlay of these two models reveals that the relative position of this smaller side chain, Thr78 in ZK177.8, to the 2′ position of the substrate ribose is nearly identical to its equivalent residue in hSAMHD1, L150, therefore remains “tight” selection against the (2′R)-2′-F and (2′R)-2′-OH nucleotides but allows hydrolysis of (2′S)-2′-OH, such as ara-CTP. This, in turn most likely explains the indistinguishable differences we observed when examining the hydrolysis of rNTP and (2′S)-2′-OH nucleotides (Fig. 3*C*). Two nucleotides, GTP and dATP, occupy the allosteric site located at the tetramer interface between each monomer (Fig. 4*B*). Therefore, there are total of eight nucleotides bound at the tetramer interfaces, in the context of a tetramer. This is in accordance with what has been seen in the core hSAMHD1 structures for the allosteric sites (AL), AL1 and AL2 (21). A careful comparison of our ZK177.8 with the available core hSAMHD1 crystallographic models reveal that the key residues interacting with the nucleotides are nearly identical (Fig. 4*D*). Structurally, such similarity also extends to sSAMHD1 and mSAMHD1, with the exception that there appear to be far fewer nucleotide-interacting residues within mSAMHD1 at the AL2-binding site (Fig. 4*D*).

**Figure 5:**
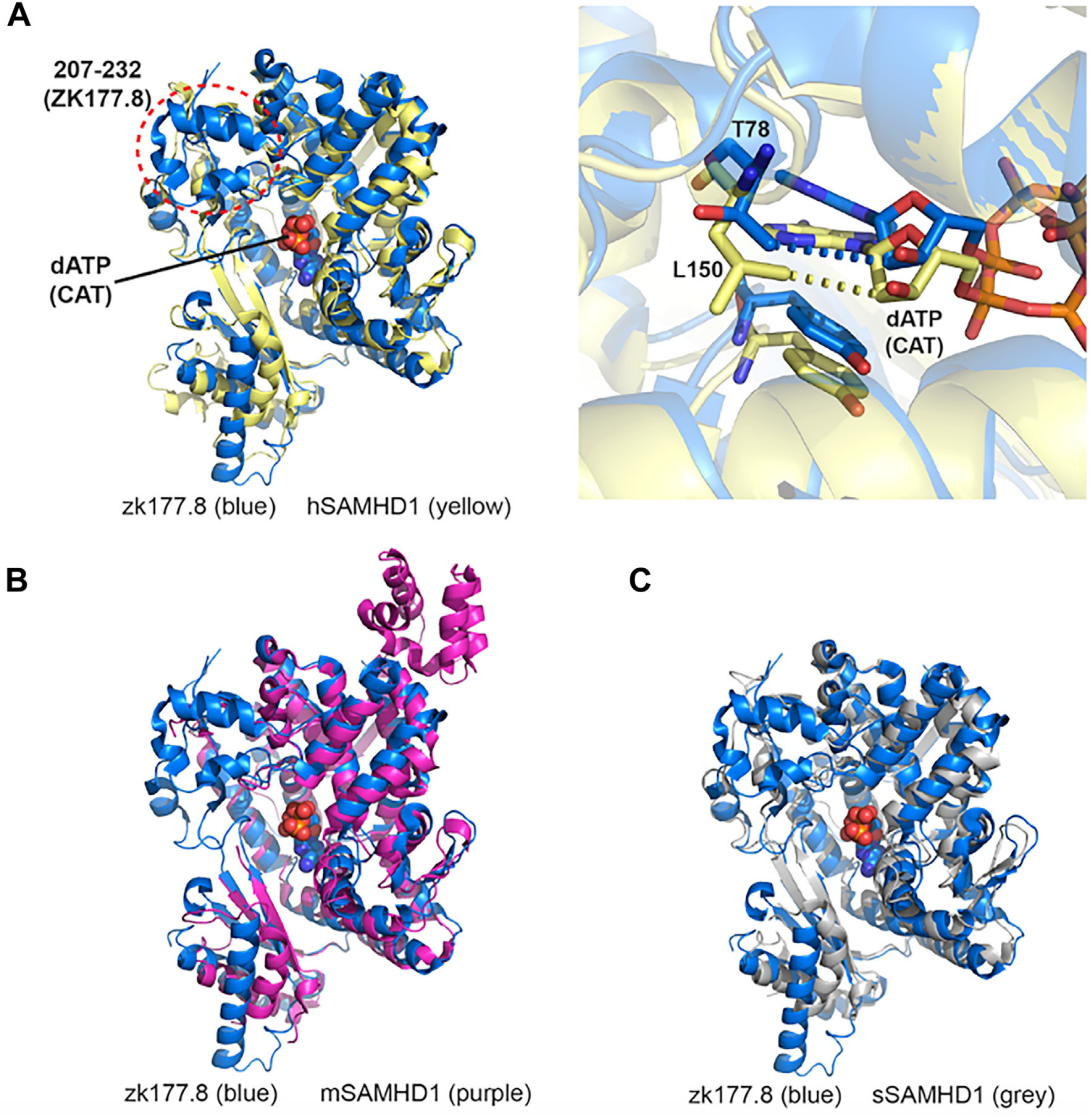
**Overall pairwise comparison of ZK177.8 and its orthologs.***A*, pairwise comparison between ZK177.8 (*blue*) and hSAMHD1 (*yellow*, Protein Data Bank [PDB] ID: 4RXP (66)), both shown in *cartoon* representation. The helix bundle mentioned in the text is indicated by the *dotted circle* (*A*, *left*). Close up of the catalytic site (*A*, *right*), with comparison of bound dATP with respect to Leu150 (ZK177.8) and Thr78 (hSAMHD1) are shown in *stick* representations. *B*, pairwise comparison between ZK177.8 and mouse SAMHD1 (*purple*, PDB ID: 6BRK (52)). *C*, pairwise comparison between ZK177.8 and pig SAMHD1 (*gray*, PDB ID: 5YHW, *unpublished*). In all figures, the location of the catalytic site is indicated by the bound dATP (ZK177.8) shown in *sphere* representation as a reference. All pairwise comparisons were performed by DaliLite, version 5 (54). SAMHD1, SAM domain and HD domain–containing protein 1.

For the overall structure, the refined model of ZK177.8^41–565^ is structurally highly homologous to the HD domain of hSAMHD1. The overlay of the two models shows high similarity in between the HD domains of ZK177.8 and hSAMHD1 (Fig. 5*A*) with the notable exception of the helix bundle (residues 207–232) that is present in ZK177.8 but not in hSAMHD1 (see the *red dotted circle* in Fig. 5*A*). The high similarity between these two models is also indicated by the pairwise comparison results from Dali server (54) with *z*-score of 46.6, RMSD of 2.3 Å, and sequence identity of 30%. This overall structural similarity was also observed with mSAMHD1 (52) and sSAMHD1 (unpublished) as shown in the overlays of these models in Figure 5*B* for mSAMHD1 and Figure 5*C* for sSAMHD1. Combined with our biochemical assays, where dGTP or GTP is required to activate the ZK177.8 for its dNTPase activity, ZK177.8 indeed most likely requires dGTP or GTP binding at the AL1 site and binding of any deoxynucleotide at the AL2 site by inducing assembly of the active tetramer as seen in hSAMHD1.

## Discussion

SAMHD1 is involved in various human diseases such as AGS neurodevelopmental genetic disorder (1, 2, 3, 4), cancers (36, 44, 51, 55), and HIV-1 infection in myeloid (6, 7, 8) cells as well as multiple cellular events, such as cell cycle progress (38), dsDNA break repair (41), DNA replication fork processing (42), and anticancer activity of araC (43, 56). In this study, since *C. elegans* has been extensively explored as animal models for various human diseases including Parkinson’s disease (57) and Alzheimer's disease (58), we explored a series of biochemical and structural validations of ZK177.8 SAMHD1 ortholog, which is required for its potential as a model for the SAMHD1-related human disorders.

A genome-wide gene knockdown survey in *C. elegans* previously reported that the reduction of the SAMHD1 ortholog ZK177.8 expression led to maternal sterility, which is a strong indication for the role of ZK177.8 in host development (47). Indeed, a series of our functional characterizations presented in this study confirm that ZK177.8 displays very similar mechanistic properties with hSAMHD1, including the dGTP/GTP-mediated activation of the dNTPase, the specificity of substrates and activators, and oligomerization. Also ZK177.8 was able to reduce cellular dNTP level elevated by the loss of hSAMHD1 in THP-1 macrophage model.

dNTP biosynthesis, which includes various cell cycle–regulated enzymes such as ribonucleotide reductase and thymidine kinase, has been extensively studied, and pharmacological targeting against these dNTP biosynthesis enzymes has been developed as anticancer agents (59). However, dNTP degradation was not considered as a key dNTP regulatory mechanism until SAMHD1 was identified as a nondividing cell–specific HIV-1 restriction factor that suppresses the viral reverse transcription step that consumes cellular dNTPs, particularly in nondividing myeloid cells including macrophages. Possibly, SAMHD1-mediated dNTP hydrolysis plays a key regulatory role in metabolic homeostasis of cellular dNTPs in nondividing cells that lack DNA synthesis and replication.

Our study of the structure of ZK177.8 shows that ZK177.8 is highly homologous to its human ortholog, hSAMHD1. Within their catalytic sites, the requirement for deoxynucleotides as their substrates is controlled by first, the presence of tyrosine residues (Tyr374 and Tyr325 in hSAMHD1 and ZK177.8, respectively). Furthermore, the sensitivity against (2′R)-2′-F and (2′R)-2′-OH nucleotides with 2′-OH group in “down” position (in comparison to reduced, yet hydrolyzable (2′S)-2′-OH nucleotides with 2′-OH group in “up” position, such as ara-CTP) has been reported with hSAMHD1, and its leucine residue (Leu150) appears to play a key role here (43). In ZK177.8, a smaller side-chain residue, threonine (Thr78) occupies at the equivalent site, indicating the selectivity profiles might be altered in ZK177.8. However, our detailed comparison between two structures shows that the respective distance between these side chains to the bound nucleotides are identical, also supporting our biochemical analysis that their sensitivity against various nucleotides with 2’-OH are indistinguishable. Interestingly, the dGTP concentration–dependent dGTPase activity comparison of hSAMHD1 and ZK177.8 proteins showed that hSAMHD1 hydrolyzes dGTP more efficiently than ZK177.8 at lower dGTP concentrations (Fig. S3). More detailed enzyme kinetic analysis with various substrates and activators will reveal the enzymatic similarities and differences between these two enzymes. Finally, ZK177.8 does not encode any identifiable SAM domain, which is dispensable for the dNTPase activity of hSAMHD1, and the role of the SAM domain in hSAMHD1 still remains unclear. Therefore, how lack of the SAM domain affects the biological roles of ZK177.8 needs to be further investigated.

Our structure of ZK177.8 supports that it is also allosterically regulated, as the identified allosteric sites, AL1 and AL2, show similar patterns of regulating residues proximal to the bound nucleotides. Within the first allosteric site, AL1, only nucleotides with guanosine base are allowed (GTP or dGTP). It is monitored by Asp137, Gln142, and Arg145 in hSAMHD1, and our structure of ZK177.8 shows that the base of GTP molecule is also tightly monitored through interactions by Asp46, Gln51, and Arg54. As in hSAMHD1, the ZK177.8 structure also shows that there are no side chains monitoring the type of ribose at AL1, in line with our biochemical analysis where ZK177.8 can be activated not only by dGTP but also by GTP. In hSAMHD1, the AL2 site requires the opposite; no discrimination against the type of base, yet no ribose with 2′-OH is allowed, proposed to be mainly because of Phe157 residue. Our structure of ZK177.8 also shows Tyr66 at its equivalent site, supporting the idea that this residue will also induce steric hindrance to prevent rNTP bindings at AL2 site.

Overall, this study demonstrates evolutionary conservation between ZK177.8 and hSAMHD1 in their enzyme functions, regulatory mechanisms, and structures as dNTPases. This biochemical study of ZK177.8 protein supports its potentials as animal models for various SAMHD1-related human disorders (AGS, infectious diseases, and cancers) and cellular events such as cell proliferation and intracellular dNTP regulation. Further investigations on the *in vivo* biological roles of ZK177.8 protein will validate its potentials as animal models of these SAMHD1-related human disorders.

## Experimental procedures

### Plasmids

Total *C. elegans* RNA was extracted from adult *C. elegans*, and ZK177.8 complementary DNA was reverse transcribed using SuperScript III Reverse Transcriptase (ThermoFisher). The full-length ZK177.8 complementary DNA was cloned into pGEX5x-3 (GE Healthcare) for protein purification, and the N–T HA-tagged ZK177.8 gene was cloned to pLVX-IRES-mCherry (Clontech) for the ZK177.8 expression in human THP-1 cells. The truncated ZK177.8 construct (residues 41–565) used in structural studies was generated by introducing the NdeI restriction enzyme site at the position corresponding to the residues 39 and 40 onto the full-length ZK177.8 by PCR amplification and cloning to pET14b. All catalytically inactive HD mutants (H134R/D135N), both full-length and truncated ZK177.8, were generated by site-directed mutagenesis.

### ZK177.8 protein expression and purification

The full-length ZK177.8 gene product (ceSAMHD1-pGEX5x-3) used in all the biochemical studies was expressed in Rosetta DE3 cells (Novagen) by inducing with 0.2 mM IPTG at absorbance of 0.5 to 0.8 for 48 h at 16 °C. Cells were harvested by centrifugation at 4000*g* for 30 min, followed by sonication in lysis buffer containing 40 mM Tris–HCl at pH 7.5, 250 mM KCl, 5% glycerol, 0.1% Triton X-100, 5 mM beta-mercaptoethanol (β-Me), 0.1 mM PMSF, and 0.5 mM benzamidine. Cleared lysate was obtained by centrifugation at 39,000*g* and applied to a GSTrap FF column (GE Healthcare) that had been equilibrated with GST binding buffer containing 50 mM Tris–HCl (pH 7.5), 10% glycerol, 250 mM KCl, and 5 mM β-Me. The column was washed for 20 column volume (CV) with the GST binding buffer, followed by 5 CV wash with increased KCl (1 M final). The column was re-equilibrated for 5 CV with protease cleavage buffer containing 100 mM NaCl and 2 mM CaCl_2_, and factor Xa (New England Biolabs) was added (50 U/CV) to the column and allowed to cleave overnight on-column at 4 °C. The protein was then eluted with the protease cleavage buffer. Fractions containing ZK177.8 protein were combined and further purified on Superdex S200 10/300 (GE Healthcare) with gel-filtration buffer containing 50 mM Tris–HCl at pH 7.5, 20% glycerol, 150 mM KCl, 1 mM β-Me, and 0.25 mM EDTA, and the fraction corresponding to the dimer of ZK177.8 (major product) was combined and flash frozen in liquid nitrogen and stored in −80 °C until use. Purity of the purified ceSAMHD1 is determined to be >95%, as judged by SDS-PAGE (Fig. S1). While some minor high molecular weight–contaminated proteins were detected, this did not interfere with its structural study.

The truncated ZK177.8 (41–565) used in the structural studies was expressed under the same condition as the full-length ZK177.8 protein, and cleared cell lysate was applied to HisTrap crude FF column (GE Healthcare) that had been equilibrated with nickel (Ni) binding buffer containing 40 mM Tris–HCl (pH 7.5), 10% glycerol, 500 mM NaCl, 5 mM β-Me, and 20 mM imidazole. The column was washed for 15 to 20 CV with the Ni binding buffer, followed by 5 CV wash with increased NaCl (2M final). The column was re-equilibrated with the Ni binding buffer, followed by elution with increased imidazole (250 mM final). The fractions containing the truncated ZK177.8 proteins were pooled and further purified on Superdex S200 16/600 (GE Healthcare) with gel-filtration buffer containing 50 mM Tris–HCl (pH 7.5), 20% glycerol, 300 mM NaCl, and 5 mM β-Me. Purified proteins were pooled and flash frozen in liquid nitrogen for storage at –80 °C until use.

### HPLC-based dNTPase assay

Reactions were performed in buffer containing 50 mM Tris–HCl (pH 7.5), 50 mM KCl, and 5 mM MgCl_2_. ZK177.8 (1 μM) was incubated with 1 mM dNTP substrates with or without 50 μM of the indicated activator (18). Reactions were performed at 21 °C for 60 min, stopped by heating at 65 °C for 10 min, and centrifuged at 14,000*g* for 5 min. Reactions were mixed with dCMP loading control (1 mM) and diluted fivefold with 12% acetonitrile (10%, final) and analyzed by anion exchange HPLC using a DNAPac PA100 Nucleic Acid Column (Thermo Scientific). The column was equilibrated with buffer containing 25 mM Tris–HCl (pH 8.0) and 0.5% acetonitrile, and products were eluted with increasing amount of ammonium chloride up to 250 mM. Elution of the products was monitored at 254 nm, and peaks were quantified using 32 Karat software (Beckman Coulter). hSAMHD1 reactions were also conducted under the same condition as ZK177.8 reaction except the incubation at 37 °C.

### TLC-based dNTPase assay

Reactions were performed with [α-^32^P]dTTP or [α-^32^P]dGTP and analyzed as described previously (16). The reactions were carried out for 60 min as described for the HPLC-based assay and applied to the TLC plate, which was analyzed using a phosphorimager (Pharos FX Plus Molecular Imager; Bio-Rad). The percent of product formed was determined using densitometry analysis by Image Lab 5.2.1 (Bio-Rad) and dividing the triphosphate product by the lane total.

### Formaldehyde cross-linking assay

Cross-linking assay to determine tetramer formation was performed as previously shown elsewhere using formaldehyde (60). Briefly, both purified ZK177.8 and hSAMHD1 were first buffer exchanged into cross-linking buffer containing 40 mM sodium–Hepes (pH 7.5), 20% glycerol, 150 mM KCl, and 1 mM β-Me. Each protein at the final concentration of 10 μM was first incubated with increasing amount of dGTP (0, 0.1, 0.4, 1, and 2 mM final for ZK177.8 and 2 mM for hSAMHD1) on ice for 30 min, in the presence of 50 mM KCl and 5 mM MgCl_2_. An equal amount of freshly prepared 1.5% formaldehyde and 1.0 M glycine solution was added, and the reaction mixture was further incubated at room temperature for 15 min. The reaction was analyzed in 4 to 15% gradient SDS-PAGE after mixing with an equal volume of 2× Laemmli buffer supplemented with β-Me but without heating.

### Cell cultures and lentivirus vector production

293FT or THP-1 cells were cultured in Dulbecco's modified Eagle's medium or RPMI, respectively, supplemented with 10% fetal bovine serum and 1% penicillin–streptomycin. Vesicular stomatitis virus G pseudotyped lentivirus was produced by transfecting 293FT cells with 25 μg pSPAX2, 25 μg pLVX-IRES-ceSAMHD1-mCherry, and 10 μg vesicular stomatitis virus G encoding plasmid. Media was changed 16 h post-transfection. Produced vectors were harvested 24 and 48 h later by centrifuging the media for 5 min at 2000 rpm and collecting the supernatant. The vectors were concentrated by ultracentrifugation at 22,000 rpm for 2 h at 4 °C.

### Vector transduction and Western blots

Undifferentiated SAMHD1 KO THP-1 cells, which were established by CRISPR–Cas in our previous study (38) were transduced with the mCherry lentiviral vectors with and without ZK177.8 expression, and the mCherry-positive cells were fluorescence-activated cell sorted at 73 h post-transduction. The sorted mCherry-positive cells were differentiated into nondividing macrophage-like cells by treatment with 100 ng/ml of PMA for 72 h.

#### Western blot analysis

Whole-cell extracts were prepared from the differentiated THP-1 cells with buffer containing 1% NP-40, 1.25% deoxycholate, 0.1% SDS, 0.1 mM DTT, and 2.5 mM PMSF. Mouse anti-HA antibody and rabbit anti-GAPDH antibody were purchased from Cell Signaling. Anti-rabbit immunoglobulin G and antimouse immunoglobulin G antibodies were purchased from Sigma.

### dNTP extraction and RT-based dNTP assay

dNTPs were extracted from PMA-differentiated THP-1 cells as described previously (13). Briefly, 2 × 10^6^ cells were washed twice with 1× Dulbecco’s phosphate-buffered saline and resuspended in 200 μl ice-cold 65% methanol. Samples were vortexed, heated at 95 °C for 3 min, and centrifuged for 3 min at 14,000 rpm. The supernatant was transferred to a new tube and dried in a CentriVap Complete Vacuum Concentrator (Labconco). Samples were stored at −80 °C. Prior to use, samples were resuspended in buffer containing 50 mM Tris–HCl (pH 8.0) and 10 mM MgCl_2_. dNTP levels in the dNTP extracts were determined using a single nucleotide incorporation assay described previously (13). Two microliters of the dNTP samples isolated from known numbers of cells were used in each reaction, and the reaction products were analyzed on a 14% urea-PAGE gel. The gel was imaged using a phosphorimager (Bio-Rad) and analyzed using Quantity One software (Bio-Rad). The percent of the primer extension product was converted to the amount of dNTP contained in the 2 μl sample as described (13).

### Crystallization of ZK177.8 (41–565) H134R/D135N

The purified His-tagged ZK177.8^41–565^ was buffer exchanged into 50 mM Tris–HCl (pH 8.0), 150 mM NaCl, 5 mM MgCl_2_, and 0.5 mM Tris(2-carboxyethyl)phosphine prior to crystallization using Superdex 75. Peak fractions were pooled, concentrated to 4.0 mg/ml, and incubated with 1 mM GTP/10 mM dATP to facilitate stable tetramer formation. Crystallization was carried out using the microbatch under oil method, using a 2:1 paraffin:silicon oil mixture over the protein mixed with crystallization buffer (0.1 M SPG buffer [pH 5.5], 20% [w/v] PEG 3350) at an 1:1 ratio. After initial crystals were obtained, large and well-diffracting crystals were obtained after successive streak seeding drops containing protein mixed with the same crystallization buffer. Crystals were transferred from their mother liquor and cryoprotected in paratone oil prior to flash freezing in liquid nitrogen.

### Structure determination and refinement

Diffraction data were collected at 100 K on cryo-cooled crystals at National Synchrotron Light Source II, beamline 17-ID-2 AMX. Data were indexed in space group P1, further integrated and scaled in HKL 2000 (61), and a polyalanine model of the hSAMHD1 HD domain monomer (PDB ID: 4BZB) was used as a search model for molecular replacement employed in Phaser (University of Cambridge) (62). The initial solution placed a dimer within the unit cell, and an additional round of molecular replacement found the remaining two copies of the monomer, resulting in the full tetramer model. The model was further improved by iterative rounds of TLS refinement with the TLS group defined as each monomer and restrained refinement in Refmac5 (63), followed by manual rebuilding of the model into the 2Fo–Fc electron density maps in Coot (Medical Research Council Laboratory of Molecular Biology) (64). Bound nucleotides were inspected and modeled manually before final refinement, resulting in a final *R*/*R*_free_ of 0.18/0.21%. Full data collection and refinement statistics can be found in Table 1.

### Statistical analyses

All measurements in this study were conducted in triplicate (n = 3). Individual values are reported in each figure, and the mean of these replicates is reflected by the bars on the graphs. Error bars in each figure represent standard deviation. All statistical analyses were conducted using two-tailed and unpaired Welch's *t* tests in Prism (GraphPad Software, Inc).

## Data availability

All data generated and analyzed during this study are included in this chapter.

## Data deposition

The atomic coordinates and structure factors have been deposited in the PDB, www.wwpdb.ord (PDB ID: 6PWY).

## Supporting information

This article contains supporting information.

## Conflict of interest

The authors declare that they have no conflicts of interest with the contents of this article.
