# Supplementary material for: Biochemical functions and structure of Caenorhabditis elegans ZK177.8 protein: Aicardi–Goutières syndrome SAMHD1 dNTPase ortholog
Source: J Biol Chem. 2023 Aug 9;299(9):105148. doi: 10.1016/j.jbc.2023.105148 (PMC10485159; doi:10.1016/j.jbc.2023.105148)
Supplement: Supporting Figures S1–S3 [file mmc1.docx]

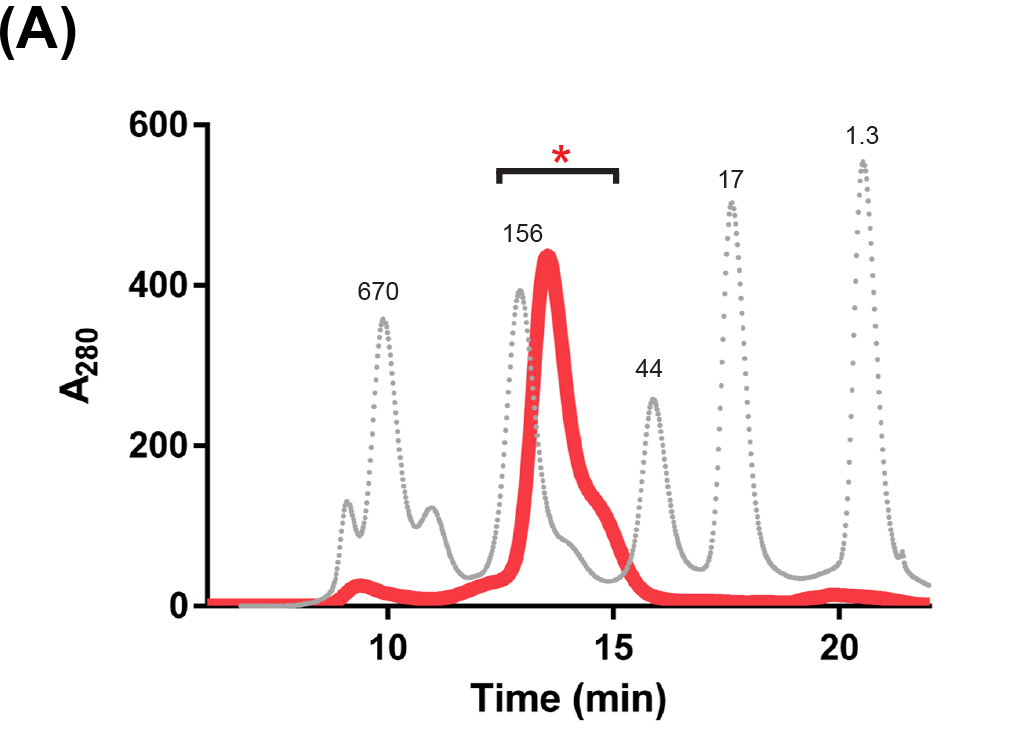


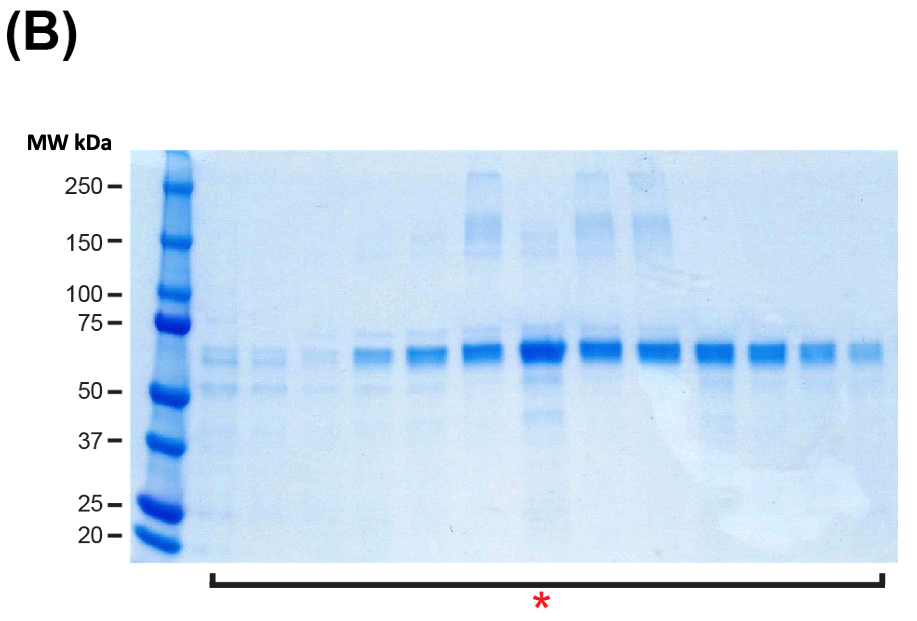


**Figure S1: Purification profile of ZK177.8 protein from HLPC.** The fractions eluted from Ni-column chromatography post the precision protease cleavage reaction (red) were pooled and applied to gel filtration chromatography (Superdex 200 10/300) as described in Experimental Procedures. The gel filtration fractions containing the protein peak equivalent to a molecular weight of the ZK177.8 dimer marked with “*” in (A) were analyzed by SDS-PAGE (B). These fractions were pooled and used for the biochemical and structural analyses. Molecular weight standards were also shown in (A) and (B). Markers: thyreoglobulin (670kDa), bovine β-globulin (156kDa), ovalbumin (44kDa), Myoglobulin (17kDa), and B12 vitamin (1.3kDa).


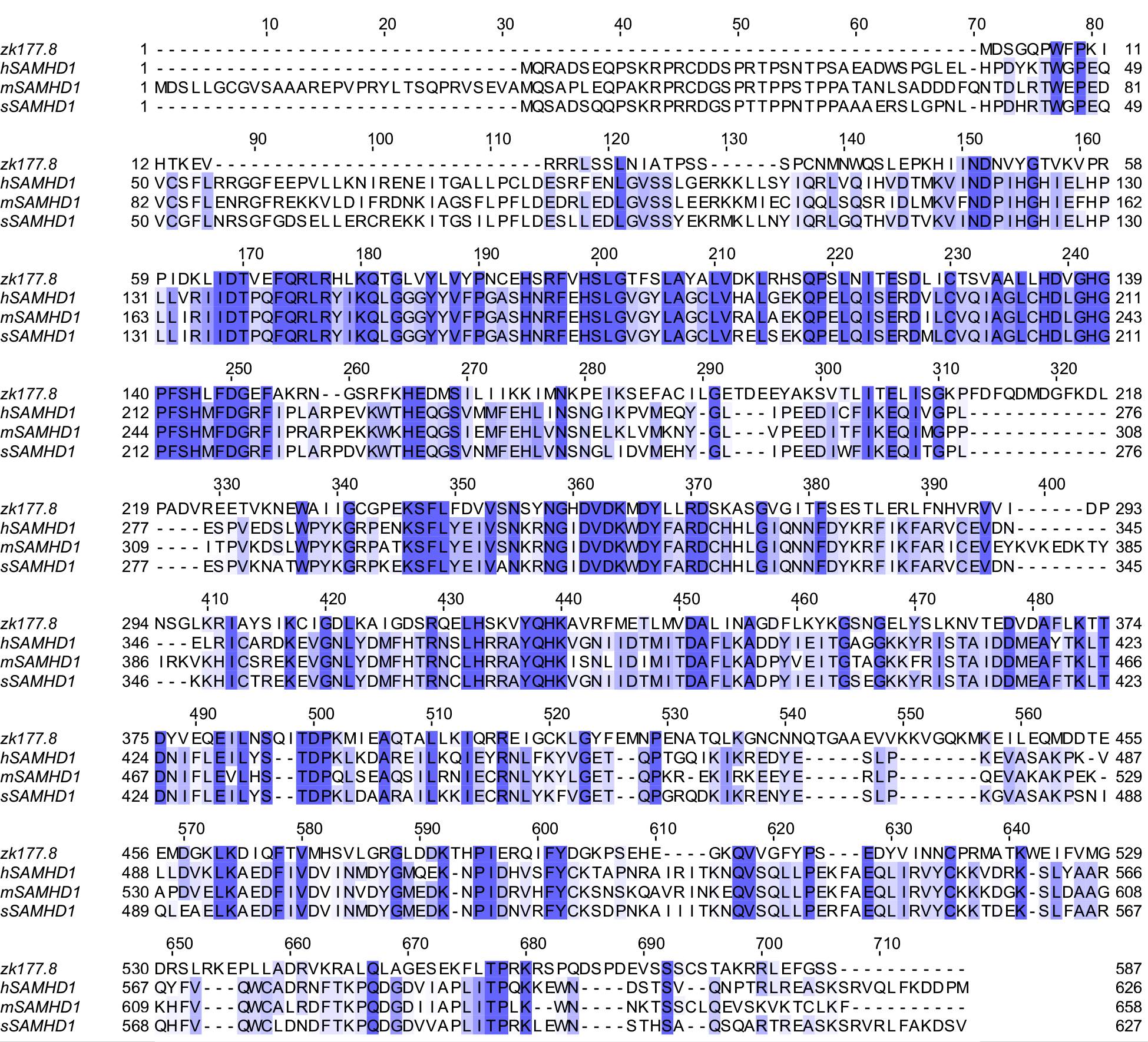


**Figure S2: Amino acid sequence comparison among human SAMHD1, mouse SAMHD1 and ZK177.8.** Conserved residues are marked with color.

**
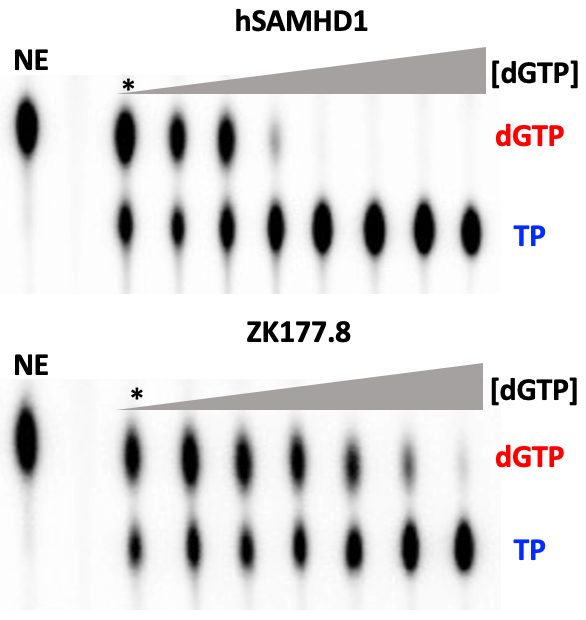
**

**Figure S3: dGTP concentration-dependent dGTPase activity of hSAMHD1 and ZK177.8 proteins.** The dGTPase activity assay of hSAMHD1 and ZK177.8 proteins was conducted and analyzed as described in Figure 2D (TLC-based analysis). The protein concentrations showing ~50% dGTP hydrolysis at the lowest dGTP concentration (“*”, 1.33nM [α-^32^P] dGTP with no cold dGTP) were used for the same reactions with increasing concentrations of excess cold dGTP (0.5, 1, 5, 25, 50,100 and 250μM). dGTP: substrate, TP: triphosphate product. NE: No enzyme
